# Effect of bacterial root symbiosis and urea as source of nitrogen on performance of soybean plants grown hydroponically for Bioregenerative Life Support Systems (BLSSs)

**DOI:** 10.3389/fpls.2015.00888

**Published:** 2015-10-26

**Authors:** Roberta Paradiso, Roberta Buonomo, Mike A. Dixon, Giancarlo Barbieri, Stefania De Pascale

**Affiliations:** ^1^Division of Plant Biology and Crop Science, Department of Agricultural and Food Sciences, University of Naples Federico IIPortici, Italy; ^2^School of Environmental Sciences, University of GuelphGuelph, ON, Canada

**Keywords:** *Glycine max* (L.) Merr., *Bradyrhizobium japonicum*, Nutrient Film Technique (NFT), root nodulation, fertigation

## Abstract

Soybean is traditionally grown in soil, where root symbiosis with *Bradyrhizobium japonicum* can supply nitrogen (N), by means of bacterial fixation of atmospheric N_2_. Nitrogen fertilizers inhibit N-fixing bacteria. However, urea is profitably used in soybean cultivation in soil, where urease enzymes of telluric microbes catalyze the hydrolysis to ammonium, which has a lighter inhibitory effect compared to nitrate. Previous researches demonstrated that soybean can be grown hydroponically with recirculating complete nitrate-based nutrient solutions. In Space, urea derived from crew urine could be used as N source, with positive effects in resource procurement and waste recycling. However, whether the plants are able to use urea as the sole source of N and its effect on root symbiosis with *B. japonicum* is still unclear in hydroponics. We compared the effect of two N sources, nitrate and urea, on plant growth and physiology, and seed yield and quality of soybean grown in closed-loop Nutrient Film Technique (NFT) in growth chamber, with or without inoculation with *B. japonicum*. Urea limited plant growth and seed yield compared to nitrate by determining nutrient deficiency, due to its low utilization efficiency in the early developmental stages, and reduced nutrients uptake (K, Ca, and Mg) throughout the whole growing cycle. Root inoculation with *B. japonicum* did not improve plant performance, regardless of the N source. Specifically, nodulation increased under fertigation with urea compared to nitrate, but this effect did not result in higher leaf N content and better biomass and seed production. Urea was not suitable as sole N source for soybean in closed-loop NFT. However, the ability to use urea increased from young to adult plants, suggesting the possibility to apply it during reproductive phase or in combination with nitrate in earlier developmental stages. Root symbiosis did not contribute significantly to N nutrition and did not enhance the plant ability to use urea, possibly because of ineffective infection process and nodule functioning in hydroponics.

## Introduction

Soybean [*Glycine max* (L.) Merr.] is a *Leguminosae* species. Similarly to other legumes, when grown in soil, soybean can obtain a certain amount of nitrogen (N), in the form of ammonium (NH_4_^+^), from natural fixation of atmospheric N_2_ by *Rhizobium* symbiotic bacteria, producing nodules on plant roots (Howard and Rees, 1996). *Rhizobium* with specific mutualistic relationship with soybean is *Bradyrhizobium japonicum* (Somasegaran and Hoben, 1994).

Biological N_2_ fixation (BNF) can make plants self-sustaining for N nutrition, avoiding the need for mineral fertilization (Salvagiotti et al., 2008). However, the nodulation process and N_2_ fixation are energetically more costly to the host than N uptake from substrate (Phillips, 1980; Ryle et al., 1984). As a consequence, plants strictly control nodulation and when a sufficient amount of usable N is available, as in complete nutrient solutions in hydroponics, an auto-regulatory circuit may inhibit root infection (Herridge et al., 1984; Abdel Wahab et al., 1996), as well as nodule establishment and development and nitrogenase activity (Eaglesham, 1989). In general, the severity of this inhibition increases with N concentration in the growth medium (Barbulova et al., 2007) and legume-bacteria symbiosis seems to be adversely affected by nitrate more than by ammonium (Svenning et al., 1996; Bollman and Vessey, 2006) and urea (Cheema and Ahmad, 2000). Different inhibitory effects of nitrate, ammonium, and urea have been observed in hydroponically grown soybean (Vigue et al., 1977; Imsande, 1988) and pea (Waterer et al., 1992; Gulden and Vessey, 1997), while results are controversial in other legumes, since NH_4_^+^ seems to repress nodulation in faba bean and white lupin (Guo et al., 1992). However, at biochemical level most of the studies on N-dependent nodulation inhibition pathways rely on effects of high NO_3_^-^ concentration (Carroll et al., 1985a; Streeter, 1985; Streeter and Wong, 1988), and only a few are focused on NH_4_^+^ (Barbulova et al., 2007) and urea (Carroll et al., 1985b) action. Beside, it is known that ammonium can be nitrified both in soil and hydroponic nutrient solutions, so even when ammonium is supplied, in some circumstances the effect may be due to nitrate (Savvas et al., 2013).

In soybean in field conditions, BNF accounts for about 50% of plant requirement on average. However, the effects of N fertilization are extremely variable, due to interactions of BNF with soil, external N sources and other factors, such as soil moisture and temperature and method of fertilizer application (Salvagiotti et al., 2008). If applied properly (e.g., slow-release, polymer coated product in deep placement, below the nodulation zone), urea is as effective as nitrate in soybean. Indeed, despite of possible volatilization losses and slower plant uptake, it allows N utilization from fertilizers with lower or no concomitant decrease in symbiotic N_2_ fixation (Cheema and Ahmad, 2000).

The project MELiSSA (Micro-Ecological Life Support System Alternative) of the ESA aims to design a closed-loop artificial ecosystem for resources regeneration, for long term manned missions in Space. This system is based on biological components such as microorganisms and higher plants, able to recycle human waste and to produce food, water and oxygen in BLSSs (Hendrickx et al., 2006). Most of the studies aiming to characterize crops production under controlled environment, in the sight of their use in BLSSs, are conducted in hydroponic (or soilless) systems with closed-loop fertigation strategy and recirculating nutrient solution (Wheeler et al., 2003).

Soybean is one of the crops selected as a candidate for cultivation in BLSSs, because of the high nutritional value of seeds, rich in proteins and lipids (De Micco et al., 2012; Palermo et al., 2012). Soybean can be grown hydroponically with complete nitrate based nutrient solutions (Wheeler et al., 2008; Paradiso et al., 2012), which are the most common in hydroponics, with positive effects on seed nutritional quality compared to open field (Palermo et al., 2012).

Urea is an important ammonium fertilizer in soil cultivation (Salvagiotti et al., 2008) Nevertheless, it is seldom used in hydroponics, where the hydrolysis to NH_4_^+^ and CO_2_ is not mediated by urease enzymes of telluric microbes and is normally negligible in water solution, at the temperature commonly used for plant growth (Warner, 1942). Partial replacement of nitrate with urea in hydroponics is reported to improve plant productivity, while reducing nitrate accumulation in leafy (Gunes et al., 1994) and fruit vegetables (Gerendas and Sattelmacher, 1997; Tan et al., 2000). Conversely, when tested as the sole N source, urea reduced the growth of these crops (Luo et al., 1993; Khan et al., 1997). However, the use efficiency of urea is influenced by plant genotype and pH and temperature of nutrient solution (Engels and Marschner, 1995), and only a few reports are available on soybean in hydroponics (Cheema and Ahmad, 2000). Evidence that urea is absorbed and relocated as an intact molecule exists for some crops, such as cereals (Mérigout et al., 2008) and tomato (Tan et al., 2000) in hydroponics, while no data seem to be available for soybean.

Resource recycling is a critical topic in Space research because of technical problems of supplying the entire quantity of resources needed (food, water, and oxygen), and managing waste in long-duration missions (Mackowiak et al., 1996; De Micco et al., 2009). In this context, liquid wastes (crew urine and wash-water) represent the dominant waste stream in BLSSs, and urea is about 85% of the recyclable N source potentially available for plant growth, being included in hygiene water (laundry and shower/hands cleansing agents) and both urine and faces of astronauts (Wydeven and Golub, 1990). Beside, using urea as source of N would reduce the volume of fertilizers to be carried in Space (including acids to buffer the typical pH fluctuations in nitrate solutions). However, there is a lack of information on the influence of urea, as an alternative N source to nitrate, on plant performance and bacteria root symbiosis in soybean grown in hydroponics with recirculating solution. Specifically, only a few works investigated the ability of soybean plants to use urea as the sole source of N in nutrient solution, and on its effect on root symbiosis with *B. japonicum* (Vigue et al., 1977; Israel and Jackson, 1982).

Within a series of experiments aiming to set up the best protocol for cultivation in BLSSs (Paradiso et al., 2014a), the objective of this study was to compare the effect of two N sources, nitrate and urea, on plant growth and physiology, and seed yield and quality of soybean, in closed-loop NFT in growth chamber, with or without inoculation with *B. japonicum*.

## Materials and Methods

### Cultivation Design, Growth Chamber Environmental Control and Hydroponic System Management

The experiment was carried out at the Controlled Environment Systems Research Facility (CESRF) of the University of Guelph (Guelph, ON, Canada).

**FIGURE 1:**
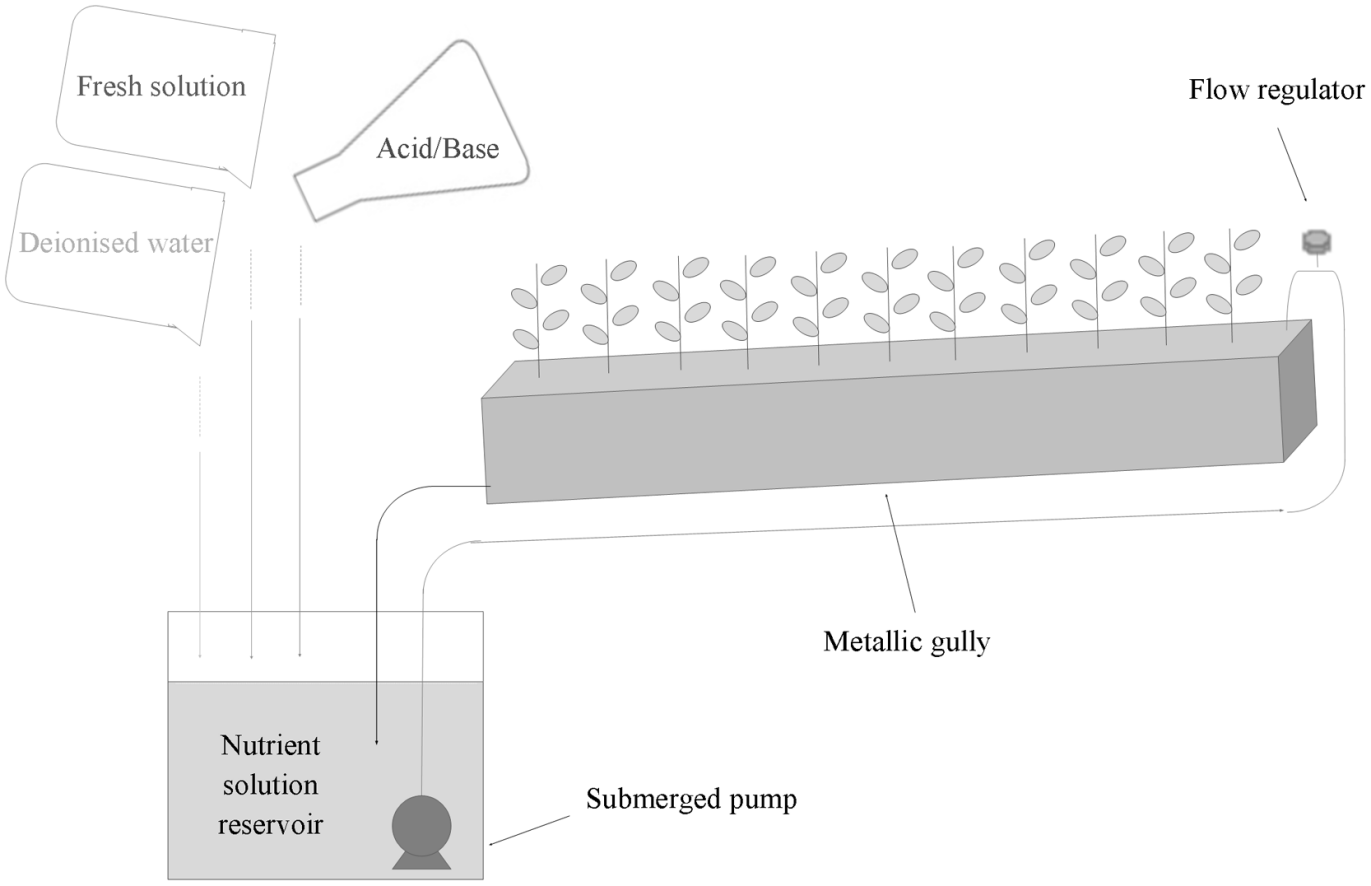
**Scheme of NFT stainless steel gully and strategy of management of the recirculating nutrient solution for the hydroponic cultivation of soybean**.

Soybean plants of the Canadian cultivar ‘OT9814’ were grown in a 15 m^2^ growth chamber, equipped with a recirculating NFT system, consisting in 12 stainless steel gullies (**Figure 1**), at a plant density of 50 plants m^-2^.

Environmental conditions were kept constant during the growing cycle. Light was provided according to a light/dark regime of 16/8 h, using fluorescent tubes (Philips Sylvania 215W Very high output Cool white T12). Tubes were mounted on mobile panels which were moved upward following the stem elongation, in order to keep the PAR at the top of the canopy at 750 μmol m^-2^ s^-1^. Details on the lighting system were described in Paradiso et al. (2014b). Set up temperatures were 26/18°C (light/dark) and relative humidity (RH) in the light time was kept within the range of 70–85%, using a fog system. The mean values recorded at the end of the experiment (122 days) were 25.2 ± 0.6°C/18.4 ± 0.4°C and 85.5 ± 9.5%, respectively (Mean ± Standard Deviation). The experiment was carried out at ambient CO_2_ concentration (370–400 ppm).

The following treatments were compared:

- two N sources in the nutrient solution: nitrate fertilizers, NO_3_ [Ca(NO_3_)_2_ and KNO_3_] vs. urea, U [CO(NH_2_)_2_];
- absence or presence of root symbiosis: no inoculation (C) vs. inoculation with *B. japonicum* strain BUS-2 (I).

Treatments were factorially combined. Three gullies, arranged in randomized blocks, were used for each treatment (11 plants per gully; 33 plants per treatment in total).

Sowing was preceded by seed sterilization (Somasegaran and Hoben, 1994). Bacterial inoculation was performed on seeds in a peat carrier solution, as reported by Vincent (1970). Non-inoculated seeds were exposed to the same environmental conditions of the inoculation protocol. Seeds were germinated in Petri dishes, on agar-based substrate. Inoculation was repeated on plantlets, at transplanting (12 DAS), in a 10% sucrose solution. Roots of control plantlets (C treatment) were dipped in the sucrose solution only.

The Hoagland solution ½S, modified by Wheeler et al. (2008) for soybean specific requirements, was used as NO_3_-based solution. The nutrient concentration was: (in mM) N 7.5, P 0.50, K 4.3, Ca 2.5, Mg 1.0, S 1.0; (in μM) Fe 60, Mn 7.4, Zn 0.96, Cu 1.04, B 7.13, Mo 0.01. A similar recipe, obtained by replacing nitrate with urea, was used as the U-based solution: (in mM) N 12.0, P 0.25, K 3.0, Ca 3.0, Mg 1.0, S 3.0, (in μM) Fe 54, Mn 7.4, Zn 0.96, Cu 1.04, B 7.13, Mo 0.05. Nutrient solution was completely replaced once (80 DAS).

Electrical conductivity and pH targets were 1.2 dS m^-1^ and 6.0 respectively, and they were controlled manually every day and adjusted three times a week. Water depletion was measured twice a week and volume and EC of recirculating solution were kept constant by adding deionised water and/or fresh solution. pH was controlled by adding HNO_3_ 0.5 M (NO_3_ solution) and H_3_PO_4_ 0.5 M (U solution) to lower pH, or KOH 0.5M to raise pH. Higher concentration of N was used in the U recipe to compensate the amount of this element added as HNO_3_ in the NO_3_-solution. Similarly, a higher quantity of P and K were used in the NO_3_ recipe to balance the addition through H_3_PO_4_ and KOH in U solution. In both the solutions, 2(*N*-Morpholino)-Ethane-Sulfonic acid (MES) 2 mM was used as a buffering agent to minimize pH fluctuations.

Fertigation was performed continuously, with one separate nutrient solution reservoir per each treatment (3 gullies). Gullies were sealed with polyethylene film to prevent evaporation. Nutrient solution was replaced at 80 DAS (68 days after transplanting), corresponding to approximately the middle of the growing cycle, which lasted 122 DAS.

### Sampling and Measurements

Plant water consumption for transpiration was determined twice a week as water depletion in each gully (11 plants per gully), assuming the water uptake unaffected by evaporation due to the sealing.

Net photosynthesis was measured using a portable gas exchange system (Li-6400, Licor Inc., Lincoln, NB, USA). Measurements were carried out on the middle leaflet of the second and third fully expanded trifoliate leaves from the top, in three plants per treatment, during the different phenological phases: vegetative growth, flowering and pod filling (33, 47, and 66 DAS, respectively). The conditions inside the leaf chamber were set at the following values: temperature 26°C, RH 60%, CO_2_ concentration 400 ppm, PAR 700 μmol m^-2^ s^-1^ (obtained with an internal lamp).

The chlorophyll content was estimated using a colorimeter (CCM-200 chlorophyll meter, Opti-Sciences, Inc.), and expressed as CCM-200 units, in 6 plants per treatment (two leaves per plant, two measurements per leaf), at flowering (47 DAS).

Growth analysis was based on non-destructive measurements of plant height and number of leaves, at 7-day intervals until the appearance of pods, and 21-day intervals during the pod-filling, on six plants per treatment. Once a month, LA was measured on three plants, using a LA meter (Li-3100, Li-Cor, Lincoln, NB, USA). Root nodulation was monitored monthly, starting 30 DAS, in terms of number of nodules per plant and individual size of nodule, determined as dry weight after oven drying to a stable weight at 60°C. At the harvest, yield was determined as g of seeds (edible biomass) on a unit area basis, at the 14% of water content. The HI was calculated as DM of seeds as fraction of the total DM of plant (excluding roots).

Chemical analysis of leaves was performed once a month, to determine the main nutrient concentration, according to the AOAC 990.03 (N) and AOAC 985.01 (K, Ca, Mg) protocols (Aoac, 1990). Analyses were carried out on DM of three plants per each combination *Nitrogen source x Root symbiosis*. Proximate composition of seeds (protein, fat, carbohydrate, and ash content) was determined by means of standard procedures AOAC (1990), on three seed samples per treatment, 200 g of fresh weight each. Proteins were calculated on the basis of total N content determined by Kjeldahl method, using 6.25 as the conversion factor from N to protein. Chemical analysis of nutrient solution were performed twice to measure the content of NO_3_- and NH_4_- nitrogen, before replacing the nutrient solution (80 DAS), and at the end of the growing cycle (122 DAS) (Aoac, 1990).

At the end of the experiment, the following indexes of resource use efficiency were calculated: WUE, as g of edible DM per liter of nutrient solution consumed; RUE, as g of edible DM per mole of PAR provided; AUE and BUE, as g of edible DM per mmole of H^+^ and mmole of OH^-^ supplied, respectively.

All data were analyzed with ANOVA and means were compared by the LSD test, at *P* = 0.05.

## Results

### Root Symbiosis and Plant Growth and Physiology

**FIGURE 2:**
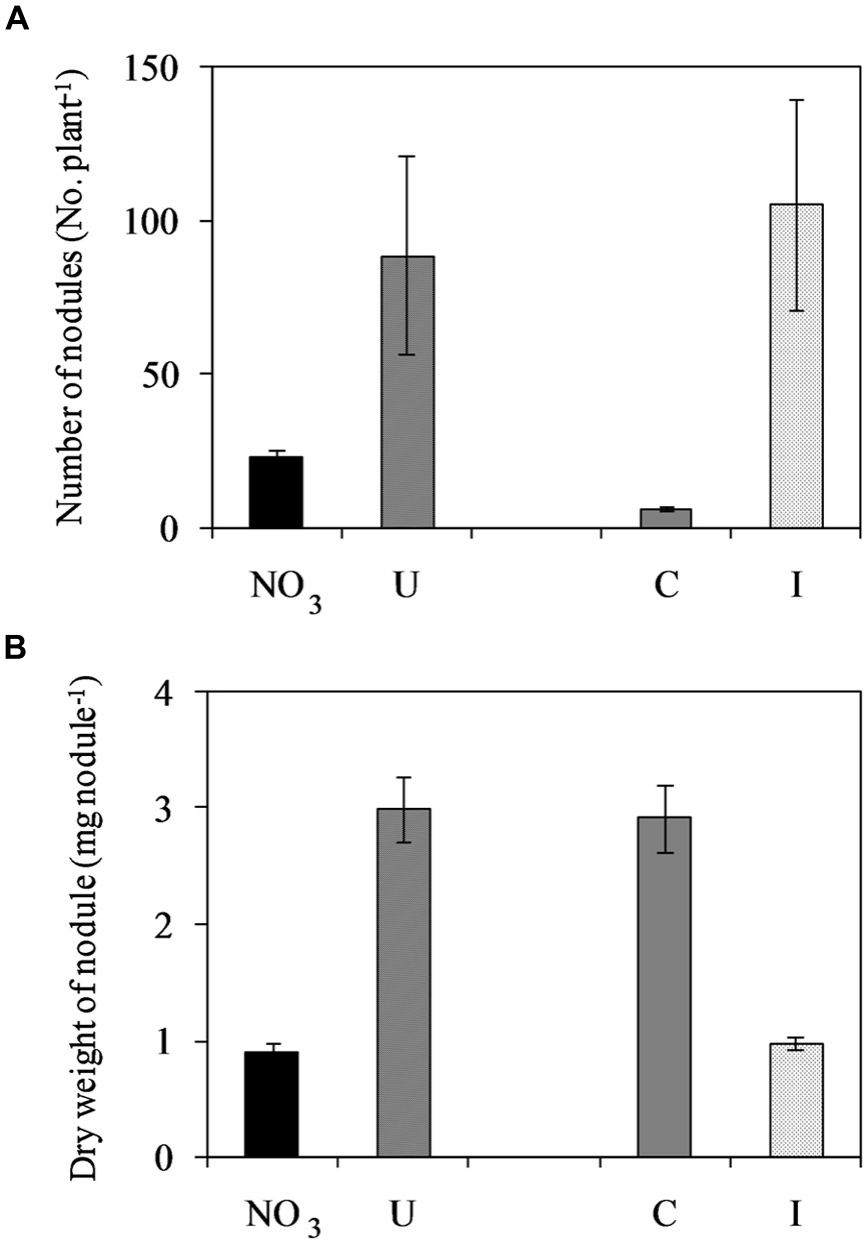
**Mean effects of nitrogen source in the nutrient solution and root inoculation with *Bradyrhizobium japonicum* on the maximum number of nodules per plant (A) and dry weight per nodule (B) in soybean grown in NFT (52 DAS, stage of late flowering – beginning of seed formation; Average values ± Standard errors, *n* = 6).** Average values of non inoculated and inoculated gullies fertigated with nitrate solution (NO_3_) and urea solution (U). Average values of gullies fertigated with nitrate and urea solutions in non inoculated control (C) and inoculated treatment (I).

Soybean plants cv ‘OT9814’ inoculated with *B. japonicum* strain BUS-2 and grown in NFT formed determinate and round nodules on roots (Supplementary Figure S1), showing a pink inner region. The number of nodules increased during vegetative growth, reached the maximum at flowering, and then decreased in the stage of pod filling (seed formation). Root nodulation was influenced by the N source in the nutrient solution. In inoculated plants fertigated with urea (6 mM in the fresh solution), the number of nodules was higher while the nodule size was smaller compared to those fed nitrate (7.5 mM in the fresh solution), during the whole experiment (Supplementary Figure S1). The maximum number of nodules was 88.8 and 22.8 per plant, respectively (**Figure 2**). Non-inoculated plants were poorly nodulated in both NO_3_- and U- solutions, however, they formed bigger nodules compared to those inoculated (**Figure 2**).

**FIGURE 3:**
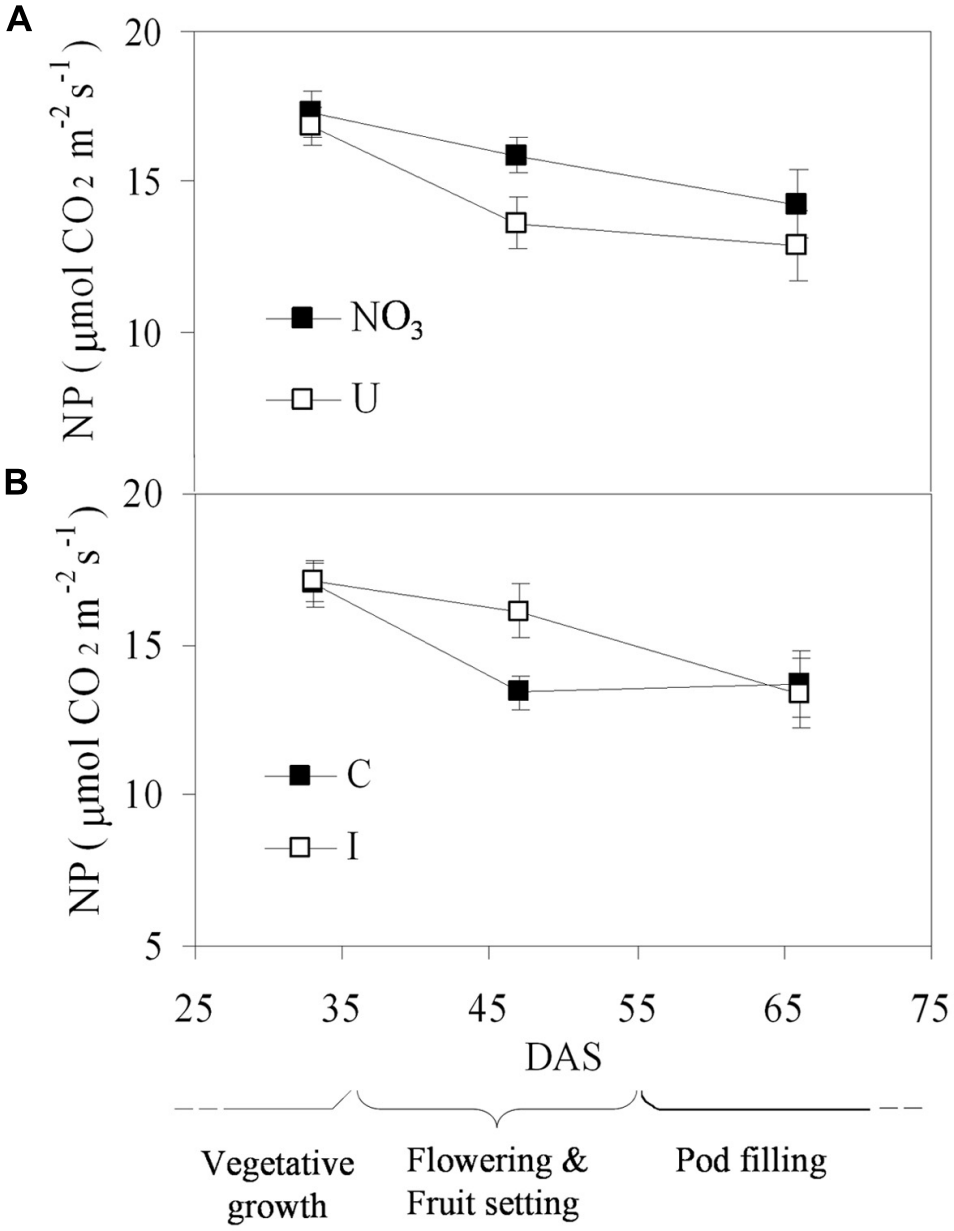
**Mean effects of nitrogen source in the nutrient solution (A) and root inoculation with *B. japonicum* (B) on the time course of NP in fully developed leaves of soybean grown in NFT (Average values ± Standard errors, *n* = 6).** Average values of non inoculated and inoculated gullies fertigated with nitrate solution (NO_3_) and urea solution (U). Average values of gullies fertigated with nitrate and urea solutions in non inoculated control (C) and inoculated treatment (I).

**FIGURE 4:**
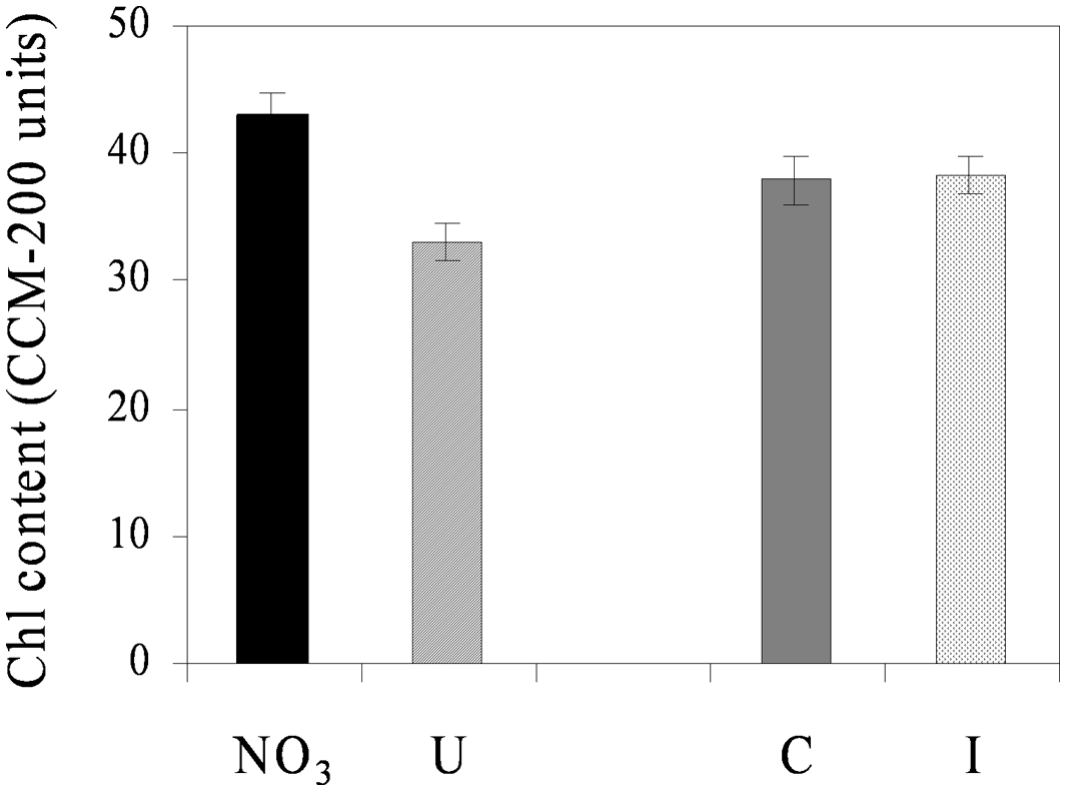
**Mean effects of nitrogen source in the nutrient solution and root inoculation with *B. japonicum* on the estimated leaf chlorophyll content (expressed as CCM-200 units) of soybean grown in NFT (52 DAS, stage of late flowering – beginning of seed formation; Average values ± Standard errors; *n* = 12).** Average values of non inoculated and inoculated gullies fertigated with nitrate solution (NO_3_) and urea solution (U). Average values of gullies fertigated with nitrate and urea solutions in non inoculated control (C) and inoculated treatment (I).

Net photosynthesis of fully developed trifoliate leaves of soybean was higher in the vegetative phase and decreased progressively during reproduction, ranging from the maximum value of 17.1 μmol CO_2_ m^-2^ s^-1^ (33 DAS) to the minimum of 13.6 μmol CO_2_ m^-2^ s^-1^ (66 DAS), on average (**Figure 3**). N sources and root symbiosis influenced the rate of photosynthesis during flowering, with lower values in urea compared to nitrate, and higher values in inoculated compared to control plants (**Figure 3**). In the same period, urea also decreased the leaf chlorophyll content (33.1 vs. 43.0 CCI units in NO_3_; **Figure 4**).

The time course of stem elongation followed a typical sigmoid pattern in all the plants, with the maximum height reached around 75 DAS. However, symptoms of physiological leaf senescence (yellowing, wilting, and curling), from the bottom to the top of the plant, started to appear around 55 DAS, and were followed by a progressive leaf falling during pod filling.

Nitrogen form influenced the plant growth and yield of soybean cv ‘OT9814’ in recirculating NFT system, while bacterial inoculation with *B. japonicum* did not determine significant effects. **Table 1** shows the maximum values of growth parameters, recorded in fully developed plants before the leaf falling. Plants fed nitrate were 120 cm high and formed 38 leaves in total, corresponding to a LA of about 2760 cm^2^ per plant, on average. Compared to nitrate, urea reduced the plant height (-25 cm) and the plant LA (-62%), reducing both the number (-20 leaves per plant) and the individual size (58.3 vs. 72.8 cm^2^) of the leaves. Accordingly, at the harvest, the total DM accumulation was significantly lower (-82%), and the seed yield decreased from 604 g per m^2^ to 40 g per m^2^. Beside, fertigation with urea significantly reduced the HI (**Table 2**).

**Table 1:** Mean effects of nitrogen source in the nutrient solution and root inoculation with *Bradyrhizobium japonicum* on growth parameters (maximum values recorded before leaf fall), seed yield and HI of soybean grown in NFT.

	Plant height (cm)	Number of leaves (n. plant^-1^)	Leaf area (cm^2^ plant^-1^)	Above ground total DM (g m^-2^)	Seed yield (g m^-2^)	HI (g seed/100 g D.M)
**N source^a^**						
NO_3_	120.3	37.9	2759	2274.9	604.1	0.41
U	94.9	18.2	1061	406.6	40.3	0.22
**Symbiosis^b^**						
C	106.8	28.2	2076	1319.7	323.0	0.29
I	108.4	27.9	1744	1361.8	321.4	0.33
N source	^∗^	^∗^	^∗^	^∗^	^∗^	^∗^
Symbiosis	ns	ns	ns	ns	ns	ns
Interaction	ns	ns	ns	ns	ns	ns

*Ns, not significant, ^∗^P ≤ 0.05. ^a^Average values of non-inoculated and inoculated gullies fertigated with nitrate solution (NO_3_) and urea solution (U); ^b^Average values of gullies fertigated with nitrate and urea solutions in non inoculated control (C) and inoculated treatment (I).*

**Table 2:** Mean effects of nitrogen source in the nutrient solution and root inoculation with *B. japonicum* on pH and EC of recirculating solution (average values after 1 day of recycling), plant cumulative water consumption and acid and base supply for pH adjustment to the target values (pH 6.0 and EC 1.2 dS/m) in soybean grown in NFT (duration of the experiment: 122 days).

	EC (EC fresh solution 1.2)	pH (pH fresh solution 6)	Water consumption (l plant^-1^)	Acid supply (mmol plant^-1^)	Base supply (mmol plant^-1^)
**N source^a^**					
NO_3_	1.6	6.5	27.2	87.7	7.8
U	1.3	5.8	6.3	15.7	22.2
**Symbiosis^b^**					
C	1.4	6.1	16.5	50.5	18.9
I	1.4	6.2	17.0	53.0	11.2
N source	^∗^	^∗^	^∗^	^∗^	^∗^
Symbiosis	ns	ns	ns	ns	^∗^
Interaction	ns	ns	ns	ns	ns

*Ns, not significant, ^∗^P ≤ 0.05. ^a^Average values of non-inoculated and inoculated gullies fertigated with nitrate solution (NO_3_) and urea solution (U); ^b^Average values of gullies fertigated with nitrate and urea solutions in non inoculated control (C) and inoculated treatment (I).*

The time course of water consumption in the 122-day growing cycle varied between the fertigation treatments, while it was unaffected by root symbiosis. In plants fed NO_3_, water consumption increased in the first weeks, as the evaporating leaf surface increased, reached the maximum around 70 DAS, and declined later because of leaf senescence and falling (maximum value 444 ml plant^-1^ per day; average value 267 ml plant^-1^ per day). Conversely, in plants fed urea it was lower and more constant during the growing cycle, because of the smaller plant size (maximum value 100 ml plant^-1^ per day; average value 61 ml plant^-1^ per day). Cumulative water consumption was 27.2 l per plant in NO_3_-treatment and 6.3 l per plant in U-treatment (**Table 2**).

The evolution of EC and pH in the recirculating nutrient solutions reflected the differences observed in plant growth and water uptake, therefore it was influenced by the N form and unaffected by the root symbiosis (**Table 2**). In general, fluctuations were small in the first weeks of growth, when the plant size was small, while they became larger and more variable in fully developed plants, and decreased progressively with plant aging. EC always tended to rise, as a consequence of plant water uptake, with higher increase in NO_3_- solution, due to the larger plant LA and the greater water consumption (**Table 2**). pH value after 1 day of recirculation increased in plants fertigated with NO_3_-solution while it slightly decreased in U-solution (**Table 2**).

The amount of acid required to control alkalinisation in the recirculating solution was higher in NO_3_- than in U-solution (+72 mmol per plant), while the cumulative supply of base to buffer acidification was higher in U-solution (+14.4 mmol per plant) (**Table 2**). The need for base addition was significantly lower in inoculated plants (**Table 2**).

Chemical analysis of recirculating U-solution revealed no trace of ammonium in the non-inoculated treatment, neither at the middle nor at the end of the experiment, while it showed the presence of ammonium in urea inoculated gullies, at both the dates (Data not shown).

### Chemical Analyses and Resources Use Efficiency

**FIGURE 5:**
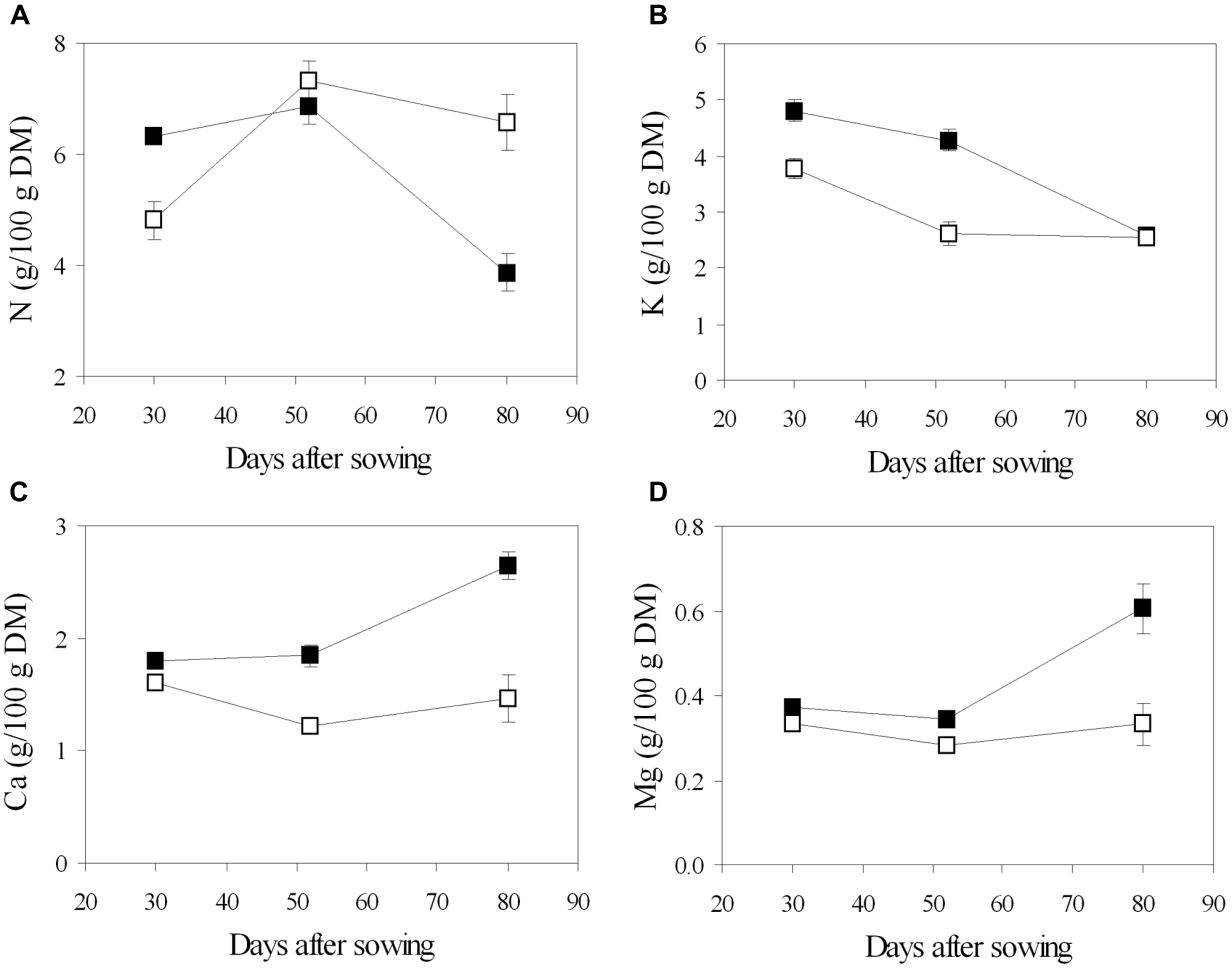
**Mean effects of nitrogen source in the nutrient on the time course of N (A), K (B), Ca (C), and Mg (D) content in leaf tissue of soybean grown in NFT (Average values ± Standard errors, *n* = 6).** Average values of non inoculated and inoculated gullies fertigated with nitrate solution (solid symbols) and urea solution (open symbol).

Chemical analysis of leaf tissues showed changes in the content of the main macronutrients throughout the phenological phases, and different values between the fertigation treatments (**Figure 5**). In general, N concentration increased from the vegetative phase (stage of 5–7 trifoliate leaves, 30 DAS) to the beginning of flowering (52 DAS), then decreased during pod filling (80 DAS) (**Figure 5**). Leaf N content was lower in urea in young plants, while it increased later and declined slower to significantly higher values in mature plants, compared to nitrate (**Figure 5**). Different pattern was observed in the main inorganic cations: K decreased from the beginning to the end of the growing cycle, regardless of the N source; Ca and Mg were constant until flowering and increased during pod filling in NO_3_-plants, while they remained almost stable along the cycle in U-plants (**Figure 5**). Root symbiosis did not alter cation composition of leaves, while it seemed to increase N content at the stage of flowering, however, the difference was found to be not significant (data not shown).

Significant effect of N form and no influence of bacterial symbiosis were also found in proximate composition of soybean seeds (**Table 3**). Protein content was higher while fat, carbohydrates and ash were lower in plants fed urea compared to nitrate.

**Table 3:** Mean effects of nitrogen source in the nutrient solution and root inoculation with *B. japonicum* on proximate composition of seeds (g/100 g DM) of soybean grown in NFT.

	Protein (%)	Fat (%)	Carbohydrate (%)	Ash (%)
**N source^a^**				
NO_3_	40.2	12.9	35.6	7.29
U	47.0	11.5	30.6	6.53
**Symbiosis^b^**				
C	43.3	12.2	33.5	7.05
I	43.9	12.3	32.8	6.76
N source	^∗^	^∗^	^∗^	^∗^
Symbiosis	ns	ns	ns	ns
Interaction	ns	ns	ns	ns

*Ns, not significant, ^∗^P ≤ 0.05.*

*^a^Average values of non-inoculated and inoculated gullies fertigated with nitrate solution (NO_3_) and urea solution (U);*

*^b^Average values of gullies fertigated with nitrate and urea solutions in non-inoculated control (C) and inoculated treatment (I).*

Fertigation of soybean with urea determined lower values of all the resource use efficiency indexes compared to nitrate (**Table 4**). WUE of seeds decreased by 70% and RUE by 93%. Similarly, relevant decrease in plants fed urea was recorded in both AUE and BUE (-62 and -98%, respectively). Plant root inoculation with *B. japonicum* improved BUE (+59%; **Table 4**).

**Table 4:** Mean effects of nitrogen source in the nutrient solution and root inoculation with *B. japonicum* on the indexes of resource use efficiency of soybean grown in NFT.

	WUE_seeds_ (g l^-1^)	RUE_seeds_ (g mol^-1^)	AUE_seeds_ (g mmol^-1^)	BUE_seeds_ (g mmol^-1^)
**N source^a^**				
NO_3_	0.44	0.29	0.32	3.78
U	0.13	0.02	0.12	0.09
**Symbiosis^b^**				
C	0.29	0.16	0.23	1.49
I	0.28	0.15	0.21	2.37
N source	^∗^	^∗^	^∗^	^∗^
Symbiosis	ns	ns	Ns	^∗^
Interaction	ns	ns	Ns	ns

*Ns, not significant, ^∗^P ≤ 0.05.*

*^a^Average values of non inoculated and inoculated gullies fertigated with nitrate solution (NO_3_) and urea solution (U);*

*^b^Average values of gullies fertigated with nitrate and urea solutions in non inoculated control (C) and inoculated treatment (I).*

## Discussion

### Effect of Nitrogen Source in the Nutrient Solution

The U-treatment, containing urea 6 mM in the fresh solution, improved root nodulation of soybean ‘OT9814’ by *B. japonicum* BUS-2, in terms of both number and size of nodules, compared to the Hoagland ½ S, containing nitrate 7.5 mM. This result confirmed stronger inhibition from nitrate compared to urea on plant–bacteria symbiosis in soybean grown in hydroponics (Imsande, 1988). Particularly, in NFT, Vigue et al. (1977) reported that nodulation decreased as NO_3_ concentration increased from 3 to 12 mM, while in urea it was similar to NO_3_ at the lowest molarity, but it did not change at increasing concentration. The same authors found that DM accumulation of nodules was maximum when urea was provided alone, rather than in any proportion with NO_3_.

The time course of NP followed a normal pattern for soybean plants, with declining rates from the vegetative to the reproductive phase. This pattern reflected the plant aging, implying an optimal nutritional status of the leaves during the former phase, and a mobilization of nutrients (i.e., N and K) from the leaves to the pods, with consequent leaf senescence, in the latter (Wheeler et al., 1996a).

Plants fertigated with urea showed lower photosynthesis during flowering compared to those fed nitrate, consistently with the lower leaf chlorophyll content. However, chemical analysis revealed lower leaf N content in urea during the vegetative phase, but similar values in the two fertigation treatments at flowering, and a slower decrease in urea during pod filling. Specifically, in vegetative phase (30 DAS), N concentration in leaves resulted higher than the optimal values for soybean in plants fertigated with the NO_3_-based Hoagland ½ S, while it was close to the minimum limit in those fed urea (reference interval 4.25–5.50 g/100 g DM; Hellal and Abdelhamid, 2013). Afterward, the rising tendency of leaf N could depend on increasing uptake of urea over time, possibly due to higher use efficiency in soybean adult plants, as described for *Arabidopsis* (Mérigout et al., 2008) and tomato (Tan et al., 2000) in hydroponics. This later availability would account for a delay in N migration from the leaves to the pods, explaining the higher leaf N content in U-plants during pod filling, as well as the lower seed yield. Indeed, remobilization of vegetative N to the developing pods is known to be critical for seed yield and quality in soybean, since over 60% of the whole-plant N can be remobilized, with a relevant contribution to seed formation (Munier-Jolain et al., 1996). Beside, N absorbed as urea is known to be less mobile than that absorbed as NO_3_^-^, contributing less to fruit and seed setting and development by translocation (Tan et al., 2000). Similarly to what concluded in tomato (Tan et al., 2000), the poor absorption, limited translocation, and slow assimilation of N can be considered the main causes of growth reduction when urea was applied as the sole N source in hydroponics in soybean seedlings, even though the ability to use urea increased in the following growth stages.

In our experiment, fertigation with urea also resulted in significantly lower uptake of essential cations (K, Ca, and Mg), causing nutrient deficiency, as observed in tomato (Ikeda and Tan, 1998). In this respect, it is known that electroneutrality in cytosol of plant tissues is strictly required for the proper cell functioning, and that organic and inorganic anions and cations contribute to the maintanance of the ionic equilibrium. NO_3_^-^ plays a fundamental role in this process due to the relevant plant requirement of N (Kirkby and Mengel, 1967). Indeed, when plants uptake NO_3_^-^, a great quantity of inorganic cations has to be uptaken, and a certain amount on hydroxil or bicarbonate has to be excreted, to counterbalance the relevant anion charge. When urea is used to replace nitrate, an undissociate, non-ionic molecule substitutes a main ionic component and, since urea is uptaken as such with no need for the plant to balance its absorption, a reduction in uptake of other ions occurs. This process also implies alterations in the osmotic homeostasis, since NO_3_ and the organic acids synthesized during its metabolisms serve as important osmolites (Gerendas and Sattelmacher, 1997). Similarly to our experiment, reduced K and Mg uptake aimed at electrochemically balancing the anion-to-cation ratio has been described in common bean fed with N-free solution, with consequent impaired growth and yield, ascribed to both N shortage and reduced cations uptake (Kontopoulou et al., 2015). In addition, in our study, it is likely that urea accumulation in recirculating solution, due to the poor plant consumption, determined toxic effects in soybean (Luo et al., 1993; Tan et al., 2000). In tomato, Ikeda and Tan (1998) found a concentration threshold associated with toxicity symptoms (12 mM). Similarly, Gerendas and Sattelmacher (1997) found toxicity symptoms in zucchini determined by urea excess due to insufficient uptake. These results indicate that the range of urea concentration in the solution tolerated by plants is narrower than that of nitrate, which varies from 14 to 18 mM depending on the species (Savvas et al., 2013). Consequently, urea can result in toxicity symptoms *per se* when provided as the exclusive N source even at relatively low level, even though plant sensitivity changes among species, depending on the rate of urease activity (particularly in roots; Luo et al., 1993; Real-Guerra et al., 2013).

Consistently with the detrimental effects on mineral nutrition and photosynthetic performance, urea as the sole source of N reduced the biomass accumulation of soybean compared to nitrate. In parallel, the smaller plant size implied a smaller photosynthesizing LA, which further limited plant growth and seed production. Also, plants fed urea showed a higher proportion of DM accumulated in inedible parts (stem, leaves, pods) and lower values of all the resource use efficiency indexes.

Soybean Canadian cv. ‘OT9814’ under nitrate fertigation gave similar seed yield but taller plants with greater LA compared to ‘Pr91m10’ (fertigated with Hoagland ½ S in NFT), selected in the MELiSSA project as the best European cultivar for BLSSs. As a consequence, the HI was lower (0.41 vs. 0.57 in ‘Pr91m10’; Paradiso et al., 2012). HI depends on both seed production and DM accumulation in inedible parts. In our experiment, LA of ‘OT9814’ fed NO_3_ was much higher compared to the values obtained for soybean in both soil and hydroponics (Paradiso et al., 2012). In this respect, ‘OT9814’ revealed to be unsuitable for food production in BLSSs, where short size and small waste production are desired, because of the limited volume available for cultivation and storage.

Nutritional analysis of seeds of ‘OT8914’ showed higher protein and lower fat content compared to the above mentioned European cv (Paradiso et al., 2012), but similar values to the American cv McCall (Wheeler et al., 1996b), both grown in NFT under nitrate fertigation. Our results in proximate composition confirmed higher nutrient content in NFT compared to soil, as reported by Palermo et al. (2012), who demonstrated that hydroponics promoted fat and dietary fiber accumulation. The different content of proteins in seeds of plants fed with the different N forms could be caused by concentration/dilution effect due to the different yield in the two treatments. Specifically, the higher protein content in seeds of urea-plants as compared to nitrate could be due to the reduced yield, as a result of the partitioning of the available reserve of N in fewer seeds.

In terms of fertigation management, the increase of EC, constantly recorded during the growing cycle in both the recirculating solutions, indicated the greater uptake of water over nutrients (Lea-Cox et al., 1999). The amplitude of this increase changed over time with plant growth and differed between the fertigation treatments, since the proportion of these uptake depends on transpiration rate and nutritional needs. Indeed, EC fluctuations were wider in adult than in young plants and under NO_3_- compared to U-fertigation, because of the larger transpirating leaf surface and the consequent higher water consumption.

In NO_3_-recipe, the passage through the root system and the plant uptake resulted in the physiological alkalinisation of recirculating solution, determined by a greater uptake of anions (mainly nitrate) over cations, and a parallel eﬄux of OH^-^ (Marschner, 1995; Ferrante et al., 2000). As a consequence, in the Hoagland solution (the most common recipe for Space-related experiments) the use of sole nitrate causes a large input of acid for pH control, which becomes itself a major source of N, resulting in N over-supply and excessive vegetative growth (Wheeler et al., 1999). Beside, NO_3_ can accumulate in plant tissues and become harmful to human health, and the quantity of acid could become considerable in BLSSs in the context of long-duration missions, and be dangerous because of its corrosive or explosive nature (Wheeler et al., 2008). Conversely, the non-ionic feature of urea and the absence of hydrolysis in hydroponics guarantees more stable values of pH (Xu et al., 2012). In our experiment the replacement of nitrate salts [Ca(NO_3_)_2_ and KNO_3_], physiologically neutral or slightly alkaline, with K_2_SO_4_, physiologically acidic, further reduced the acid requirements, while determined the need for small quantities of KOH for pH adjustment.

Water use efficiency, RUE and AUE calculated in plants fed nitrate compared favorably to those obtained in soybean cultivars by (Wheeler et al., 2003, 2008), when using the Hoagland recipe, while no data seem to be reported for use efficiency indexes of this crop fertigated with urea.

### Effect of Bacterial Root Symbiosis

Limited infection by *B. japonicum* occurred in non-inoculated plants of soybean grown in growth chamber. Contamination between inoculated and control treatment by *Rhizobium* in controlled environment has been reported also by Ralston and Imsande (1983).

Root inoculation with *B. japonicum* BUS-2 increased NP of soybean ‘OT8914’ during the stage of flowering. In soybean, two metabolic changes which reduce the efficiency of the photosynthetic machinery over time are documented: decreasing in root growth and functioning, which slows xylem flow of water and nutrients and hormonal translocation (indirectly restricting the rate of photosynthesis), and mobilization of N from leaves to developing seeds (which directly lowers photosynthetic output) (Imsande, 1988). However, it is known that the severity of these processes is alleviated when soybean plants are nodulated, as in our experiment, since bacterial fixation increases the supply of usable N (i.e., ureides), stimulating plant photosynthesis compared to non-inoculated plants (Imsande and Schmidt, 1998). This hypothesis is in accordance with the slight increase in leaf N content found at flowering in inoculated plants compared to those not incoulated.

Despite the positive effect on photosynthesis at flowering, bacterial symbiosis did not enhance significantly plant nitrogen uptake and did not improve growth and yield of soybean plants and proximate composition of seeds, regardless of the chemical form of N in the nutrient solution. This result may be related to the particular root environment in NFT, where most of the roots are constantly exposed to the continuous motion of nutrient solution, that could adversely affect the establishment of bacterial infection (attaching to the root hair; Tittabutr et al., 2007), and submerged in anoxic or low oxygen conditions, which can obstruct the infection mechanism (Goormachtig et al., 2004) and the N_2_-fixing activity of nodules (Bomfeti et al., 2013). This latter hypothesis is supported by results of our previous experiment on the same soybean cv, comparing NFT and cultivation on rockwool. Indeed, the use of rockwool resulted in better nodulation and plant performance respect to the sole liquid medium, thanks to the protective function on bacteria and the more favorable conditions for root development and functioning in the solid substrate (Paradiso et al., 2014b). In addition, it is known that the availability of N (which is typical of hydroponic nutrient solutions) retards and limits the nodule formation, and that this inhibition is stronger when external N is supplied since the early stages of plant growth (Caetano-Anollés and Gresshoff, 1991; Ferguson et al., 2010). On the other hand, inoculation of legumes results in efficient nodulation and BNF only after a period of growth (e.g., 3–5 weeks for *Rhizobium tropici* in common bean, fed with N-free nutrient solution; Kontopoulou et al., 2015). Consequently, an adequate supply of N during this period is essential to feed plants, and also to prevent the parallel reduction in cations uptake determined by the absence of NO_3_. At the same time, this quantity should minimize the inhibitory effect of N on bacteria. Specifically, since a certain concentration of mineral N in the root zone, defined as starter N, stimulates the nodule establishment and activity, further research should be addressed to identify this critical concentration for soybean cultivars selected for hydroponic cultivation under controlled environment, as this value is known to vary with genotype and growth conditions (Waterer and Vessey, 1993; Gan et al., 2004; Liu et al., 2011).

Root inoculation with *B. japonicum* did not affect the management of the recirculating solution (volume adjustment and acid supply), with the exception of the base addition, which was significantly lower compared to non-inoculated control, because of bacterial production of NH_3_ which caused alkalinisation (Somasegaran and Hoben, 1994).

The absence of ammonium, registered in the non-inoculated U-solution at the middle of and at the end the experiment confirmed the lack of urea hydrolysis in hydroponics, as reported by Kirkby and Mengel (1967) in tomato grown in aerated nutrient solution.

## Conclusion

At the environmental conditions of our experiment, the Canadian cultivar of soybean ‘OT8914,’ grown in closed-loop NFT with nitrate-based nutrient solution, showed agronomical traits unsuitable for cultivation in BLSS (excessive size and inedible biomass production).

Urea as the sole source of N increased root nodulation by *B. japonicum*, but it drastically reduced the plant growth and seed yield of soybean compared to nitrate. These detrimental effects were determined by nutrient deficiency, because of low plant use efficiency of urea as such, and consequent N deficiency in the early stages of development, and reduced cations uptake throughout the whole growing cycle.

Inoculation with *B. japonicum* did not enhance significantly plant nitrogen uptake, and ultimately did not improve plant performance of soybean grown in NFT, regardless of the N source in the nutrient solution. The absence of significant effects of root symbiosis on plant could be due to non-optimal infection process and nodule functioning in NFT, and the consequent scarce contribution of bacteria to plant N nutrition, as both N_2_ fixation and enzymatic hydrolysis of urea.

In our experimental conditions, urea was not suitable as N fertilizer for soybean in closed-loop NFT. However, the ability to use urea increased from young to adult plants, suggesting the possibility to apply it during reproductive phase or, even in earlier developmental stages, in combination with nitrate. Further studies should be addressed to investigate the starter N concentration or variable nitrate/urea ratio in the nutrient solution in the different phenological phases, in order to optimize plant nutrition while promoting bacterial activity. For instance, combining small doses of urea with nitrate in the early stages could help nodulation, by limiting the inhibiting effect of nitrate, while enhancing plant nutrition by providing a readily available source of N, when the contribution of N_2_ fixation is limited and the plant efficiency in urea utilization is still low. Afterward, the effect of the sole urea could be investigated in later stages, when the root nodules are well established and the plant use efficiency and tolerance to toxicity are higher.

## Conflict of Interest Statement

The authors declare that the research was conducted in the absence of any commercial or financial relationships that could be construed as a potential conflict of interest.

## Supplementary material

FIGURE S1 **Differences in root system and nodule size in inoculated plants of soybean grown in NFT as function of the nitrogen source in the nutrient solution: nitrate [left] and urea [right], (52 DAS, stage of beginning of flowering – beginning of seed formation)**.

## Abbreviations

AUE: acid use efficiency
BLSSs: bioregenerative life support systems
BNF: biological nitrogen fixation
BUE: base use efficiency
Cv: cultivar (cultivated variety)
DAS: days after sowing
DM: dry matter
EC: electrical conductivity
ESA: European Space Agency
Half strength: ½ S
HI: Harvest Index
LA: leaf area
NFT: Nutrient Film Technique
NP: net photosynthesis
PAR: photosynthetically active radiation
RUE: radiation use efficiency
WUE: water use efficiency

## References

[B1] Abdel Wahab A. M. Zahran H. H. Abd-Alla M. H. (1996). Root-hair infection and nodulation of four grain legumes as affected by the form and the application time of nitrogen fertilizer. *Folia Microbiol.* 41 303–308. 10.1007/BF02814705

[B2] Aoac (1990). *Official Methods of Analysis*, 14th Edn. Washington, DC: Association of Official Analytical Chemists.

[B3] Barbulova A. Rogato A. D’Apuzzo E. Omrane S. Chiurazzi M. (2007). Differential effects of combined N sources on early steps of the Nod factor-dependent transduction pathway in *Lotus japonicus*. *Mol. Plant Microbe Interact.* 20 994–1003. 10.1094/MPMI-20-8-0994 17722702

[B4] Bollman M. I. Vessey J. K. (2006). Differential effects of nitrate and ammonium supply on nodule initiation, development, and distribution on roots of pea (*Pisum sativum*). *Can. J. Bot.* 84 893–903. 10.1139/b06-027

[B5] Bomfeti C. A. Ferreira P. A. A. Carvalho T. S. De Rycke R. Moreira F. M. S. Goormachtig S. (2013). Nodule development on the tropical legume *Sesbania virgata* under flooded and non-flooded conditions. *Plant Biol.* 15 93–98. 10.1111/j.1438-8677.2012.00592.x 22672666

[B6] Caetano-Anollés G. Gresshoff P. M. (1991). Plant genetic control of nodulation in legumes. *Annu. Rev. Microbiol.* 45 345–382. 10.1146/annurev.mi.45.100191.002021 1741618

[B7] Carroll B. J. McNeil D. L. Gresshoff P. M. (1985a). Isolation and properties of soybean [*Glycine max* (L.) Merr.] mutants that nodulate in the presence of high nitrate concentrations. *Proc. Natl. Acad. Sci. U.S.A.* 82 4162–4166. 10.1073/pnas.82.12.4162 16593577 PMC397955

[B8] Carroll B. J. McNeil D. L. Gresshoff P. M. (1985b). A supernodulation and nitrate-tolerant symbiotic (nts) soybean mutant. *Plant Physiol.* 78 34–40. 10.1104/pp.78.1.34 16664203 PMC1064671

[B9] Cheema Z. A. Ahmad A. (2000). Effects of urea on the nitrogen fixing capacity and growth of grain legumes - Review article. *Int. J. Agric. Biol.* 2 388–394.

[B10] De Micco V. Aronne G. Colla G. Fortezza R. De Pascale S. (2009). Agro-biology for bioregenerative life support systems in long-term Space missions: general constraints and the Italian efforts. *J. Plant Interact.* 4 241–252. 10.1080/17429140903161348

[B11] De Micco V. Buonomo R. Paradiso R. De Pascale S. Aronne G. (2012). Soybean cultivar selection for Bioregenerative Life Support Systems (BLSSs): theoretical selection. *Advan. Space Res.* 49 1415–1421. 10.1111/plb.12056

[B12] Eaglesham A. R. J. (1989). Nitrate inhibition of root-nodule symbiosis in doubly rooted soybean plants. *Crop Sci.* 29 115–119. 10.2135/cropsci1989.0011183X002900010027x

[B13] Engels C. Marschner H. (1995). “Plant uptake and utilization of nitrogen,” in *Nitrogen Fertilization in the Environment*, ed. Bacon P. (New York, NY: CRC Press), 41–70.

[B14] Ferguson B. J. Indrasumunar A. Hayashi S. Lin M. H. Lin Y. H. Reid D. E. (2010). Molecular analysis of legume nodule development and autoregulation. *Invited Exp. Rev. J. Integr. Plant Biol.* 52 61–76. 10.1111/j.1744-7909.2010.00899.x 20074141

[B15] Ferrante A. Malorgio F. Pardossi A. Serra G. Tognoni F. (2000). Growth, flower production and mineral nutrition in gerbera (*Gerbera jamesonii* H. Bolus) plants grown in substrate culture with and without nutrient solution recycling. *Advan. Hortic. Sci.* 14 99–106.

[B16] Gan Y. Stulen I. van Keulen H. Kuiper P. J. C. (2004). Low concentrations of nitrate and ammonium stimulate nodulation and N2 fixation while inhibiting specific nodulation (nodule DW g-1 root dry weight) and specific N2 fixation (N2 fixed g-1 root dry weight) in soybean. *Plant Soil* 258 281–292. 10.1023/B:PLSO.0000016558.32575.17

[B17] Gerendas J. Sattelmacher B. (1997). Significance of N source (urea vs NH4NO3) and N supply for growth, urease activity and nitrogen metabolism of zucchini (*Cucurbita pepo* convar. giromontiina). *Plant Soil* 196 217–222. 10.1023/A:1004297807151

[B18] Goormachtig S. Capoen W. Holsters M. (2004). Rhizobium infection: lessons from the versatile nodulation behaviour of water-tolerant legumes. *Trends Plant Sci.* 9 518–522. 10.1016/j.tplants.2004.09.005 15501175

[B19] Gulden R. H. Vessey J. K. (1997). The stimulating effect of ammonium on nodulation in *Pisum sativum* L. is not long lived once ammonium supply is discontinued. *Plant Soil* 195 195–205. 10.1023/A:1004249017255

[B20] Gunes A. Post W. N. K. Kirkby E. A. Aktas M. (1994). Influence of partial replacement of nitrate by aminoacid nitrogen or urea in the nutrient medium on nitrate accumulation in NFT grown winter lettuce. *J. Plant Nutr.* 17 1929–1938. 10.1080/01904169409364855

[B21] Guo R. Silsbury J. H. Graham R. D. (1992). Effect of four nitrogen compounds on nodulation and nitrogen fixation in faba bean, white lupin and medic plants. *Aust. J. Plant Physiol.* 19 501–508. 10.1071/PP9920501

[B22] Hellal F. A. Abdelhamid M. T. (2013). Nutrient management practices for enhancing soybean (*Glycine max* L.) production. *Acta Biol. Colomb.* 18 239–250.

[B23] Hendrickx L. De Wever H. Hermans V. Mastroleo F. Morin N. Wilmotte A. (2006). Microbial ecology of the closed artificial ecosystem MELiSSA (Micro-Ecological Life Support System Alternative): reinventing and compartmentalizing the Earth’s food and oxygen regeneration system for long-haul space exploration missions. *Res. Microbiol.* 157 77–86. 10.1016/j.resmic.2005.06.014 16431089

[B24] Herridge D. F. Roughley R. J. Brockwell J. (1984). Effect of rhizobia and soil nitrate on the establishment and functioning of the soybean symbiosis in the field. *Aus. J. Agric. Res.* 35 149–161. 10.1071/AR9840149

[B25] Howard J. B. Rees D. C. (1996). Structural basis of biological nitrogen fixation. *Chem. Rev.* 96 2965–2982. 10.1021/cr9500545 11848848

[B26] Ikeda H. Tan X. (1998). Urea as an organic nitrogen source for hydroponically grown tomatoes in comparison with inorganic nitrogen sources. *Soil Sci. Plant Nutr.* 44 609–615. 10.1080/00380768.1998.10414484

[B27] Imsande J. (1988). Enhanced nitrogen fixation increases net photosynthetic output and seed yield of hydroponically grown soybean. *J. Exp. Bot.* 39 1313–1321. 10.1093/jxb/39.9.1313

[B28] Imsande J. Schmidt J. M. (1998). Effect of N source during soybean pod filling on nitrogen and sulfur assimilation and remobilization. *Plant Soil* 202 41–47. 10.1023/A:1004313326745

[B29] Israel D. W. Jackson W. A. (1982). Ion balance, uptake and transport processes in N2-fixing and nitrate- and urea- dependent soybean plants. *Plant Physiol.* 69 171–178. 10.1104/pp.69.1.171 16662153 PMC426169

[B30] Khan N. K. Watanabe M. Watanabe Y. (1997). “Effect of different concentrations of urea with or without nickel on spinach (*Spinacia oleracea* L.) under hydroponic culture,” in *Plant Nutrition for Sustainable Food Production and Environment*, eds Ando T. Fujita K. Mae T. Matsumoto H. Mori S. Sekiya J. (Tokyo: Kluwer Academic Publisher), 85–86.

[B31] Kirkby E. A. Mengel K. (1967). Ionic balance in different tissues of the tomato plant in relation to nitrate, urea, or ammonium nutrition. *Plant Physiol.* 42 6–14. 10.1104/pp.42.1.6 16656486 PMC1086483

[B32] Kontopoulou C. K. Giagkou S. Stathi E. Iannetta P. M. Savvas D. (2015). Responses of hydroponically-grown common bean fed with N-free nutrient solution to root inoculation with N2-fixing bacteria. *HortScience* 50 597–602.

[B33] Lea-Cox J. D. Stutte G. W. Berry W. L. Wheeler R. M. (1999). Nutrient dynamics and pH/charge balance relationships in hydroponic solutions. *Acta Hortic.* 481 241–249. 10.17660/ActaHortic.1999.481.25

[B34] Liu Y. Wu L. Baddeley J. A. Watson C. A. (2011). Models of biological nitrogen fixation of legumes. A review. *Agron. Sustain. Dev.* 31 155–172. 10.1051/agro/2010008

[B35] Luo L. Lian Z. H. Yan X. L. (1993). Urea transformation and the adaptability of three leafy vegetables to urea as a source of nitrogen in hydroponic culture. *J. Plant Nutr.* 16 797–812. 10.1080/01904169309364575

[B36] Mackowiak C. L. Garland J. L. Sager J. C. (1996). Recycling crop residues for use in recirculating hydroponic crop production. *Acta Hortic.* 440 19–24. 10.17660/ActaHortic.1996.440.4 11541570

[B37] Marschner H. (1995). *Mineral Nutrition of Higher Plants.* New York, NY: Academic Press, 651.

[B38] Mérigout P. Lelandais M. Bitton F. Renou J. P. Briand X. Meyer C. (2008). Physiological and transcriptomic aspects of urea uptake and assimilation in *Arabidopsis* plants. *Plant Physiol.* 147 1225–1238. 10.1104/pp.108.119339 18508958 PMC2442537

[B39] Munier-Jolain N. G. Ney B. Duthion C. (1996). Termination of seed growth in relation to nitrogen content of vegetative parts in soybean plants. *Euro. J. Agron.* 5 219–225. 10.1016/S1161-0301(96)02025-4

[B40] Palermo M. Paradiso R. De Pascale S. Fogliano V. (2012). Hydroponic cultivation improves the nutritional quality of soybean and its products. *J. Agric. Food Chem.* 60 250–255. 10.1021/jf203275m 22168253

[B41] Paradiso R. Buonomo R. De Micco V. Aronne G. Palermo M. Barbieri G. (2012). Soybean cultivar selection for Bioregenerative Life Support Systems (BLSSs): hydroponic cultivation. *Advan. Space Res.* 50 1501–1511. 10.1111/plb.12056

[B42] Paradiso R. De Micco V. Buonomo R. Aronne G. Barbieri G. De Pascale S. (2014a). Soilless cultivation of soybean for Bioregenerative Life Support Systems (BLSSs): a literature review and the experience of the MELiSSA Project - Food characterization Phase I. *Plant Biol.* 16 69–78. 10.1111/plb.12056 23889907

[B43] Paradiso R. Buonomo R. Dixon M. A. Barbieri G. De Pascale S. (2014b). Soybean cultivation for Bioregenerative Life Support Systems (BLSSs): the effect of hydroponic system and nitrogen source. *Advan. Space Res.* 53 574–584. 10.1016/j.asr.2013.11.024

[B44] Phillips D. A. (1980). Efficiency of symbiotic nitrogen fixation in legumes. *Annu. Rev. Plant Physiol.* 31 29–49. 10.1146/annurev.pp.31.060180.000333 PMC542628 16660105

[B45] Ralston J. Imsande J. (1983). Nodulation of hydroponically grown soybean plants and inhibition of nodule development by nitrate. *J. Exp. Bot.* 34 1371–1378. 10.1093/jxb/34.10.1371

[B46] Real-Guerra R. Staniskçuaski F. Regina Carlini C. (2013) “Soybean urease: over a hundred years of knowledge,” in *A Comprehensive Survey of International Soybean Research - Genetics, Physiology, Agronomy and Nitrogen Relationships*, 1st Edn., ed. Board J. (Rijeka: Intech), 317–339.

[B47] Ryle G. J. A. Amott R. A. Powell C. E. Gordon A. J. (1984). N2 fixation and the respiratory costs of nodules, nitrogenase activity, and nodule growth and maintenance in Fiskeby soyabean. *J. Exp. Bot.* 35 1156–1165. 10.1093/jxb/35.8.1156

[B48] Salvagiotti F. Cassman K. G. Specht J. E. Walters D. T. Weiss A. Dobermann A. (2008). Review Nitrogen uptake, fixation and response to fertilizer N in soybeans: a review. *Field Crops Res.* 108 1–13. 10.1016/j.fcr.2008.03.001

[B49] Savvas D. Passam H. (2002) *Hydroponic Production of Vegetables and Ornamentals* Athens: D. S. Embryo Publications, 463

[B50] Savvas D. Gianquinto G. P. Tüzel Y. Gruda N. (2013). “Soilless culture,” in *Good Agricultural Practices for Greenhouse Vegetable Crops. Principles for Mediterranean Climate Areas*, eds Food and Agriculture Organization of the United Nations (FAO), (Rome: FAO, Plant Production and Protection Paper 217), 303–354.

[B51] Somasegaran P. Hoben H. J. (1994). *Handbook for Rhizobia: Methods in Legume-Rhizobium Technology.* New York, NY: Springer-Verlag, 450.

[B52] Streeter J. (1985). Nitrate Inhibition of Legume Nodule Growth and Activity. II. Short Term Studies With High Nitrate Supply. *Plant Physiol.* 77 325–328. 16664052 10.1104/pp.77.2.325 PMC1064513

[B53] Streeter J. Wong P. P. (1988). Inhibition of legume nodule formation and N2 fixation by nitrate. *Crit. Rev. Plant Sci.* 7 1–23. 10.1080/07352688809382257

[B54] Svenning M. M. Junttila O. Macduff J. H. (1996). Differential rates of inhibition of N2 fixation by sustained low concentrations of NH4+ and NO3- in northern ecotypes of white clover (*Trifolium repens* L.). *J. Exp. Bot.* 47 729–738. 10.1093/jxb/47.6.729

[B55] Tan X. W. Ikeda H. Oda M. (2000). The absorption, translocation, and assimilation of urea, nitrate or ammonium in tomato plants at different plant growth stages in hydroponic culture. *Sci. Hortic.* 84 275–283. 10.1016/S0304-4238(99)00108-9

[B56] Tittabutr P. Payakapong W. Teaumroong N. Singleton P. W. Boonkerd N. (2007). Growth, survival and field performance of bradyrhizobial liquid inoculant formulations with polymeric additives. *ScienceAsia* 33 69–77. 10.2306/scienceasia1513-1874.2007.33.069

[B57] Vigue J. T. Harper J. E. Hageman R. H. Peters D. B. (1977). Nodulation of soybeans grown hydroponically on urea. *Crop Sci.* 17 169–172. 10.2135/cropsci1977.0011183X001700010044x

[B58] Vincent J. M. (1970). *A Manual for the Practical Study of the Root-Nodule Bacteria.* Oxford: Blackwell Scientific.

[B59] Warner R. C. (1942). The kinetics of the hydrolysis of urea and of arginine. *J. Biol. Chem.* 142 705–723.

[B60] Waterer J. G. Vessey J. K. (1993). Nodulation response of autoregulated or NH4+-inhibited pea (*Pisum sativum*) after transfer to stimulatory (low) concentrations of NH4+. *Physiol. Plant.* 88 460–466. 10.1111/j.1399-3054.1993.tb01360.x

[B61] Waterer J. G. Vessey J. K. Raper C. D. Jr. (1992). Stimulation of nodulation in field peas (*Pisum sativum*) by low concentrations of ammonium in hydroponic culture. *Physiol. Plant.* 86 215–220. 10.1034/j.1399-3054.1992.860205.x 11537678

[B62] Wheeler R. M. Mackowiak C. L. Sager J. C. Knott W. M. Berry W. L. (1996a). Proximate composition of CELLS crops grown in NASA’s biomass production chamber. *Advan. Space Res.* 18 43–47. 10.1016/0273-1177(95)00860-H 11538813

[B63] Wheeler R. M. Mackowiak C. L. Stutte G. W. Sager J. C. Yorio N. C. Ruffe L. M. (1996b). NASA’s biomass production chamber: a testbed for bioregenerative life support studies. *Advan. Space Res.* 18 215–224. 10.1016/0273-1177(95)00880-N 11538800

[B64] Wheeler R. M. Mackowiak C. L. Stutte G. S. Yorio N. C. Ruffe L. M. Sager J. C. (2003). Crop production for advanced life support systems. Observations from the Kennedy Space Center breadboard project. *NASA Tech. Mem.* 58 211184.

[B65] Wheeler R. M. Mackowiak C. L. Stutte G. S. Yorio N. C. Ruffe L. M. Sager J. C. (2008). Crop productivities and radiation use efficiencies for bioregenerative life support. *Advan. Space Res.* 41 706–713. 10.1016/j.asr.2007.06.059

[B66] Wheeler R. M. Sager J. C. Berry W. L. Mackowiak C. L. Stutte G. W. Yorio N. C. (1999). Nutrient, acid and water budgets of hydroponically grown crops. *Acta Hortic.* 481 655–662. 10.17660/ActaHortic.1999.481.78

[B67] Wydeven T. Golub M. A. (1990). Generation rates and chemical composition of waste streams in a typical crewed space habitat. *NASA Tech. Mem.* 102799.

[B68] Xu G. Fan X. Miller A. J. (2012). Plant nitrogen assimilation and use efficiency. *Annu. Rev. Plant Biol.* 63 153–182. 10.1146/annurev-arplant-042811-105532 22224450

