# Supplementary figures and images for: Effect of bacterial root symbiosis and urea as source of nitrogen on performance of soybean plants grown hydroponically for Bioregenerative Life Support Systems (BLSSs)
Source: Front Plant Sci. 2015 Oct 26;6:888. doi: 10.3389/fpls.2015.00888 (PMC4620399; doi:10.3389/fpls.2015.00888)

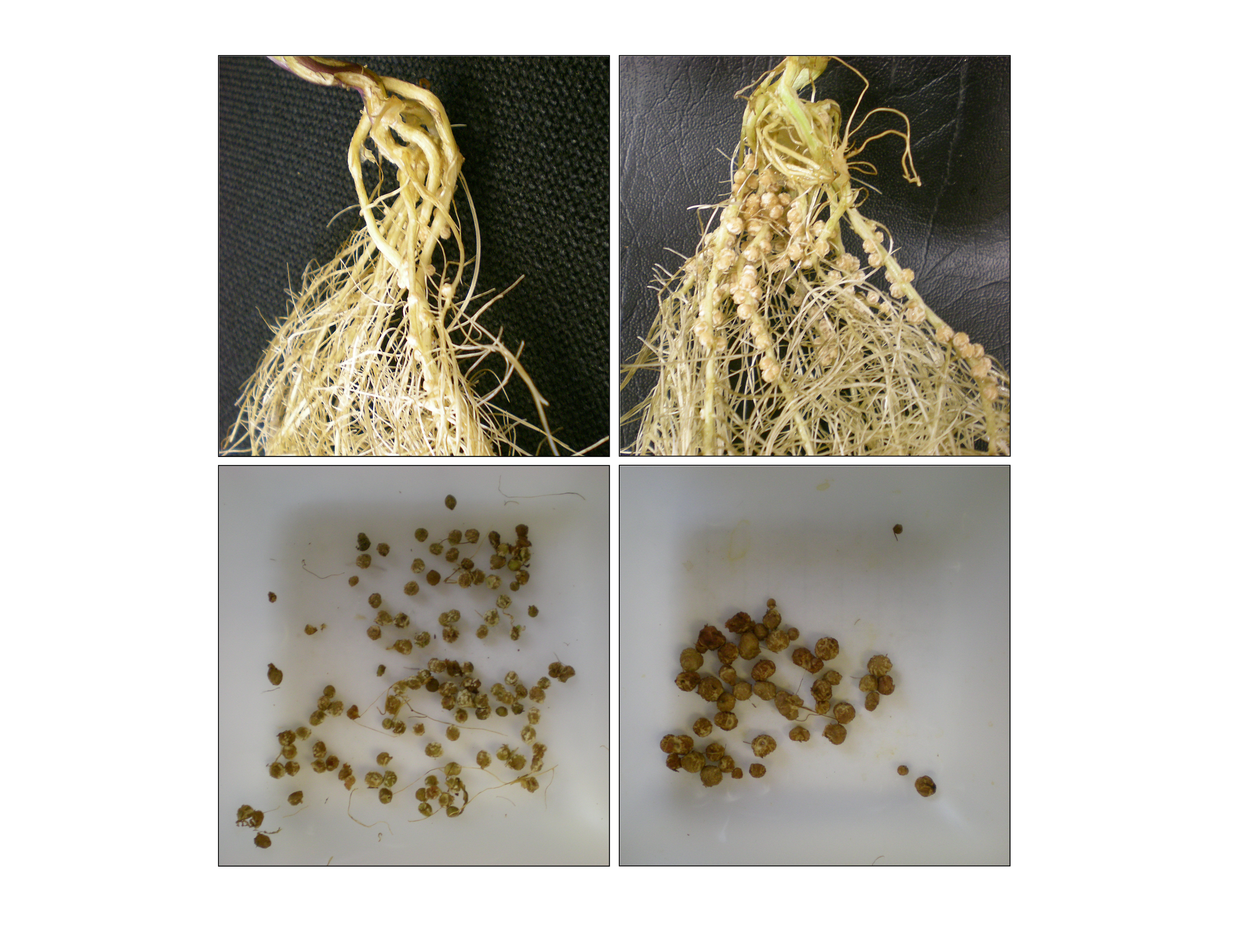

Supplement: FIGURE S1 — Differences in root system and nodule size in inoculated plants of soybean grown in NFT as function of the nitrogen source in the nutrient solution: nitrate [left] and urea [right], (52 DAS, stage of beginning of flowering – beginning of seed formation). [file Image_1.TIF]
